# Performance of a One-Dimensional Model of Wave-Driven Nearshore Alongshore Tracer Transport and Decay with Applications for Dry Weather Coastal Pollution

**DOI:** 10.1021/acs.est.2c08656

**Published:** 2023-09-22

**Authors:** Elizabeth Brasseale, Falk Feddersen, Xiaodong Wu, Amity G. Zimmer-Faust, Sarah N. Giddings

**Affiliations:** † Scripps Institution of Oceanography, 9500 Gilman Dr., La Jolla, California 92093, United States; ‡ School of Oceanography, Shanghai Jiao Tong University, 1954 Huashan Rd., Shanghai 200030, China; ¶ The Nature Conservancy, 830 S Street, Sacramento, California 96811, United States; § Southern California Coastal Water Research Project, 3535 Harbor Blvd Suite 110, Costa Mesa, California 92626, United States

**Keywords:** nearshore, pollution, modeling, water quality, waves

## Abstract

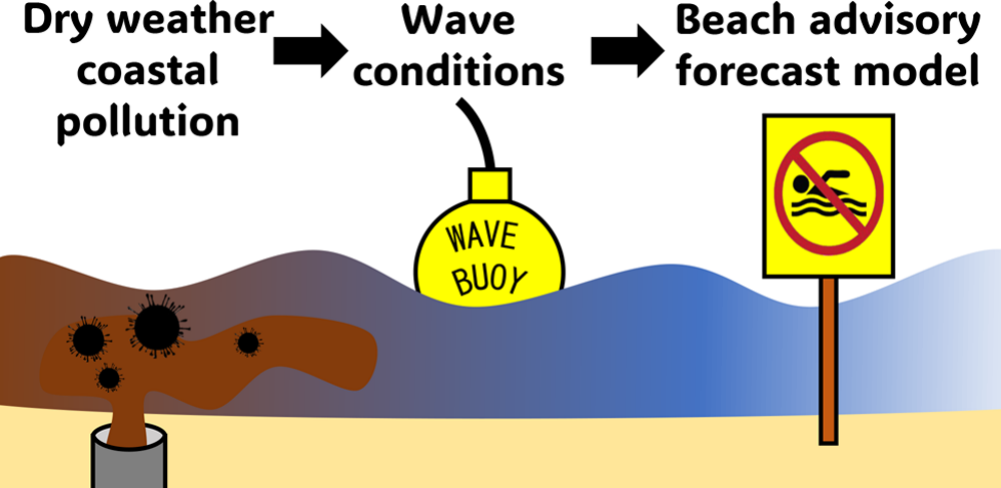

Dry weather pollution sources cause coastal water quality problems that are not accounted for in existing beach advisory metrics. A 1D wave-driven advection and loss model was developed for a 30 km nearshore domain spanning the United States/Mexico border region. Bathymetric nonuniformities, such as the inlet and shoal near the Tijuana River estuary mouth, were neglected. Nearshore alongshore velocities were estimated by using wave properties at an offshore location. The 1D model was evaluated using the hourly output of a 3D regional hydrodynamic model. The 1D model had high skill in reproducing the spatially averaged alongshore velocities from the 3D model. The 1D and 3D models agreed on tracer exceedance or nonexceedance above a human illness probability threshold for 87% of model time steps. 1D model tracer was well-correlated with targeted water samples tested for DNA-based human fecal indicators. This demonstrates that a simple, computationally fast, 1D nearshore wave-driven advection model can reproduce nearshore tracer evolution from a 3D model over a range of wave conditions ignoring bathymetric nonuniformities at this site and may be applicable to other locations.

## Abstract 2

Dynamic models complement *in situ* sampling for effective coastal water quality prediction. A 1D model is demonstrated to reproduce results and address drawbacks of a 3D hydrodynamic model.

## Introduction

**Figure 1:**
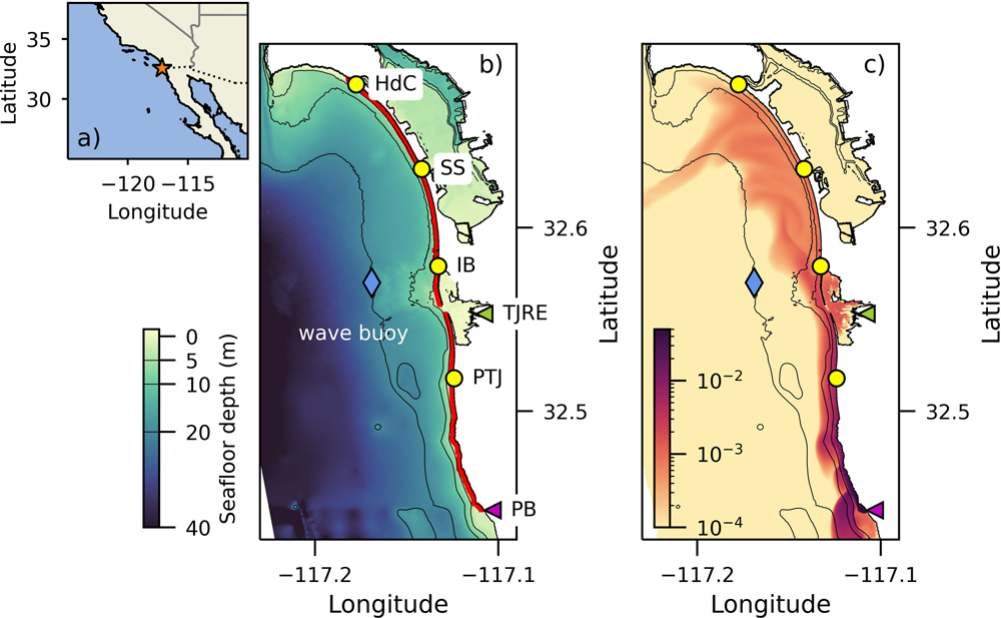
(a) Regional map with study area indicated (star) along the United States–Mexico border (dotted line). (b) SD Bight model domain with annotated landmarks. Color indicates bathymetry. The red line highlights the 29 km stretch of coastline represented in the 1D model. Magenta triangle indicates the source of wastewater to the surf zone at Punta Bandera (PB). Yellow circles represent popular recreational beaches: Playas Tijuana (PTJ), Imperial Beach (IB), Silver Strand Beach (SS), and Hotel del Coronado (HdC). Blue diamond is location of the CDIP Imperial Beach nearshore wave buoy. The green triangle indicates the head of the Tijuana River estuary (TJRE). (c) Snapshot of surface dye concentrations on a logarithmic scale on July 11, 2017, 12:00:00, when a plume from PB was transported up the coast during a long-duration south swell. Model bathymetry contoured in (b) and (c) at 5, 10, and 20 m isobaths.

Polluted nearshore waters cause gastrointestinal illness in surfers and swimmers through accidental ingestion of waterborne pathogens.^1^ Water pollution originates from nonpoint sources, such as urban and agricultural runoff after rain, and point sources, such as wastewater infrastructure failure.^2^ The San Antonio de los Buenos Wastewater Treatment Plant (SABWTP) is an example of a point source of minimally treated sewage in the United States (US)/Mexico (MX) border region. Of the 50 million gallons per day (mgd) outflow from SABWTP, treatment capacity is only 15 mgd and the remaining 35 mgd are untreated.^3^ The SABWTP outfall discharges into a coastal stream that terminates onto the beach near Punta Bandera (PB), 10 km south of the United States–Mexico border. The coastline of the San Diego Bight has over 30 km of mostly straight, sandy beach with bathymetric irregularities only near the Tijuana River Estuary (TJRE) (Figure 1). On a straight coastline, pollution point sources along the beach can contaminate nearshore waters tens of kilometers away because tracer, i.e., passively transported material, is transported alongshore efficiently and exported offshore slowly.^4−7^

In San Diego county, beach advisories are issued when fecal indicator bacteria (FIB) are found in weekly beach water quality sampling or after rainfall.^8^ However, FIB testing is not a sufficient indicator of the likelihood of illness for beach goers.^9^ FIB decay faster than other pathogens that live in wastewater and cause illness in swimmers, such as human norovirus.^10^ Rainfall is also an incomplete indicator, as dry weather runoff is increasingly recognized to have a disproportionate effect on urban coastal water quality.^11^ Inadequate wastewater treatment plant infrastructure, as is the case for SABWTP, can be a large source of dry weather runoff. Microbial source tracking during dry weather has found evidence of pollution at the shoreline 20 km north of PB.^12^ To capture the impacts of dry weather pollution sources like SABWTP outflow, existing beach advisory criteria should be supplemented with dynamical modeling.

Existing dynamic models of wastewater plume transport in the San Diego–Tijuana border region have drawbacks. A plume tracker model advects particles released from the TJRE mouth, PB, and the South Bay ocean outfall using high-frequency radar (HFR) currents to make daily water quality predictions.^13^ Among other issues, HFR does not sample within 1 km of shore, where pollution plumes are often located.^7,14^ HFR therefore cannot resolve the relevant nearshore processes to accurately estimate plume transport.^15^ A hydrodynamic model of the coastal ocean near San Diego, USA, and Tijuana, MX, that resolves both the shelf and the nearshore and tracks plumes from both TJRE and PB (hereafter “SD Bight model”) was built by coupling an ocean model to a wave model using the COAWST framework.^14,16^ However, the SD Bight model is computationally expensive (a year of model output requires weeks of runtime on a supercomputer cluster) and currently exists as a hindcast. Conversion to an operational forecast would require significant funding and effort.

An alternative solution is a nearshore model, which is appropriate for dry weather runoff water quality prediction because the input (e.g., SABWTP outflow), dynamics (wave-driven advection), and desired output (shoreline exposure to wastewater pathogens) are all located nearshore. Alongshore forcing is dominated by wave-breaking which can be estimated from an offshore wave buoy.^17−19^ Previous nearshore water quality models have reduced the problem to 1D wave-driven advection on an alongshore-uniform grid by cross-sectionally averaging tracer concentrations and alongshore transport. The models from these studies were tuned to recreate observations to derive mixing parameters and scales of biological and physical controls on water quality.^4,7,20,21^ Pathogen decay and offshore transport are represented as tracer loss from the 1D domain.^20^ Operationally, such models are many orders of magnitude faster than a full 3D hydrodynamic regional model, which is able to produce a year of model output in seconds. A 1D model is also easier to validate, automate, and use to produce forecasts because it is forced by a single data set (offshore waves). Therefore, a 1D model would be practical for daily water quality forecasts as well as efficient ensemble studies including historical and future climate predictions.

Although previous studies have demonstrated that a tuned 1D model can recreate observations, the predictive power of such a model beyond a tuning period remains to be shown. Here we test whether a 1D model with a reduced domain and reduced physics can predict nearshore tracer from a 3D hydrodynamic model after a tuning period and investigate whether similar methods can reproduce genetic marker sampling results targeting the SABWTP plume. The region of interest is a 30 km stretch of coastline from the SABWTP outflow at PB to Hotel del Coronado (HdC) (Figure 1). Comparison with a realistic 3D hydrodynamic model will demonstrate how well regional nearshore transport can be modeled, neglecting inner shelf circulation and reducing physics to wave forcing using wave properties at a single offshore source. The drawbacks of existing dynamic models, including lack of resolution of relevant processes,^13^ computational expense,^14^ and lack of calibration across different hydrodynamic conditions,^7^ are well-documented obstacles to the implementation of dynamic models for real-time water quality prediction.^22^ The 1D model developed here offers a solution to these challenges. While we are testing this 1D model in a particular region with known water quality problems, we expect the results to be applicable broadly to the skill of 1D wave-driven advection models for the transport of other tracers (e.g., sediment, plankton, or microplastics) and other similar, relatively straight coastlines.

## Materials and Methods

In this study, we compared the nearshore output of a regional hydrodynamic model with the output of a 1D reduced physics nearshore model. The first year of hydrodynamic model output, December 12, 2016, to December 31, 2017, was used as a tuning period to capture the seasonal variation in wave forcing. The next two years, from January 1, 2018, to December 25, 2019, were used to evaluate the 1D model performance. The tuning period had similar wave and nonwave forcing to the two model evaluation years.

### 3D Realistic SD Bight Model

The SD Bight model grid covers a 30 km stretch of coastline from 32.45 N (south of PB) to 32.75 N (around Point Loma) and extends 10 km offshore (Figure 1b). The SD Bight model has been used in other recent studies investigating the transport of tracers across the surf zone and inner shelf in the US/MX border region.^14,16,23−25^ The model uses the COAWST (Coupled-Ocean-Atmosphere-Wave-Sediment-Transport) modeling system.^26,27^ The SD Bight model couples Regional Ocean Modeling Systems (ROMS), a 3D hydrostatic ocean model with terrain-following vertical coordinates,^28^ with the Simulating WAves Nearshore (SWAN) model.^29^ The resulting model resolves surf zone, estuary, and shelf dynamics. The SD Bight model uses realistic atmospheric forcing (e.g., wind, heating, atmospheric pressure) from NOAA/NAM, tides, and regional river flow. The oceanic boundary conditions (temperature, salinity, sea surface height, and currents) were generated by a series of three one-way-nested parent grids.^14^ There are 10 vertical levels. The horizontal grid is rectangular and telescopic, such that the horizontal resolution is highest in the surf zone near the TJRE mouth (8 m) and lower offshore over the shelf (110 m). Model output was saved hourly to resolve tides. SD Bight model hindcasts included a dye tracer with a constant decay rate to simulate the evolution of pathogens in an untreated wastewater plume (Figure 1c). The dye tracer was input to the model at PB, the location of the SABWTP outfall, at a concentration of 0.7 to match the fraction of untreated sewage in SABWTP outflow.^3^ Horizontal tracer diffusivity was prescribed as 0.5 m^2^/s. Complete details of the model implementation and validation are in Wu et al.^14^

The nearshore was here defined from the 5 m isobath (contoured in Figure 1) to the shoreline, spanning the surf zone and a portion of the inner shelf. The 5 m isobath was chosen because it contains the offshore edge of the surf zone for all wave heights observed during the simulation period. This is the region typically used by surfers and swimmers who could be harmed by exposure to sewage. The location of the 5 m isobath and the shoreline were found for every time step to capture tidal variation. One nearshore cross-section could represent fewer than 10 or more than 100 SD Bight model grid cells depending on the local seafloor slope and horizontal resolution. Average nearshore dye and alongshore velocity were extracted from the SD Bight model from PB to HdC (red line in Figure 1b). The alongshore distance from PB, *y*, was calculated following the shoreline, defined such that positive *y* is to the right when facing the sea (roughly north). Dye and velocity were cross-sectionally averaged within the nearshore region. Velocity vectors were then rotated from grid coordinates to local alongshore and cross-shore coordinates using shorenormal angles estimated from the model grid, which were consistent with current principal axes. Velocity varied in magnitude and sign within the nearshore domain, creating localized convergence or divergence associated with offshore exchange. For comparison with 1D model alongshore velocity estimated from a sole wave buoy location, nearshore alongshore velocity was domain-averaged (from PB to HdC) and offshore exchange was represented as a uniformly distributed monotonic loss. Domain-averaged nearshore alongshore velocity from the SD Bight model will be referred to as *v̅*_C_(*t*), while cross-sectional-averaged nearshore alongshore velocity and dye will be referred to as *v*_C_(*t*, *y*) and *C*_C_(*t*, *y*), respectively. Cross section-averaged variables were interpolated onto a regularly spaced grid.

### Nearshore 1D Tracer Advection/Loss Model

Here we describe our 1D tracer advection/loss model for a nearshore dye tracer transported alongshore by wave-driven currents with loss due to physical (i.e., offshore export of dye from the nearshore region) and biological (i.e., pathogen die off) processes, hereafter, “the 1D model”. Similar 1D models of dye evolution have been used in studies that consider the transport of waterborne pathogens along beaches,^20,21^ in lagoons,^30^ and in streams.^31,32^ The 1D model solves
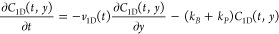
1where *y* is the alongshore coordinate, *t* is time, *C*_1D_ is the dye concentration, *v*_1D_ is the alongshore current, and *k*_*P*_ and *k*_*B*_ are constant loss terms parametrizing physical and biological processes, respectively, that reduce nearshore dye concentration. Both *v*_1D_ and loss terms (*k*_*P*_ and *k*_*B*_) are assumed alongshore-uniform, and shoreline curvature is neglected. Alongshore diffusivity was neglected (see Supporting Information).

The first loss parameter, *k*_*B*_, represents the inverse time scale of pathogen die-off. The 1D model used a 10-day e-folding time scale, *k*_*B*_ = 1.6 × 10^–6^ s^–1^, to match the prescribed dye behavior in the SD Bight model^14,16^ corresponding to the mortality of norovirus.^33^ The estimated mean e-folding time scales for other common wastewater pathogens in seawater range from less than 1 day (for *Campylobacter*) to one month or more (for *Giardia*).^33^

The second linear loss parameter *k*_*P*_ represents the cross-shelf tracer exchange between the nearshore region and the inner shelf. The *k*_*P*_ parameter may be thought of as an exchange velocity, *u*_ex_, divided by the cross shore distance from the shoreline to boundary between the nearshore and the shelf, *L*.^7,34^ This cross-shelf exchange is often driven by rip currents in observations^5,34−36^ and models.^34,37,38^ Here exchange between the surf zone and inner shelf was parametrized as a monotonic decay of nearshore tracer. We calculated *k*_*P*_ by subtracting *k*_*B*_ from the total rate of dye loss, i.e. the time-averaged dilution of dye as a function of distance from the source scaled by the root-mean-square of velocity, *V*_*RMS*_ = 0.1 ms^–1^. The resulting *k*_*P*_ = 1.3 × 10^–5^ s^–1^, consistent with estimates from nearshore observations.^7^ The relative importance of physical export to biological inactivation is organism specific.^20^ Here, export dominates, as the norovirus inactivation rate is an order of magnitude smaller than *k*_*P*_.

Nearshore alongshore advection, *v*_1D_, is assumed to be driven solely by wave-breaking. On a long, straight coastline, when wind stress is negligible (as in this region), the alongshore momentum balance in the nearshore is dominated by the cross-shore gradient of the forcing from breaking waves and bottom stress,^39,40^
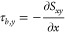
2where τ_*b*,*y*_ is the bottom stress in the alongshore direction, *S*_*xy*_ is the off-diagonal component of the radiation stress, and *x* is the cross-shore coordinate. Because *S*_*xy*_ is conserved until breaking, the relevant wave properties can be estimated at an offshore wave buoy. The alongshore current (averaged over several wave periods), *v*_1D_, can be found using the small angle and weak current approximation for bottom stress (valid when *v* ≪*u*′, where *u*′ is the cross-shore orbital velocity),^41^
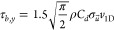
3where ρ is the density of seawater, *C*_*d*_ is a dimensionless drag coefficient used to fit *v*_1D_ to *v̅*_C_, and σ_*u⃗*_ is the variance of *u⃗*, the full velocity vector including orbital velocities. Combining eqs 2 and 3, expanding σ_*u⃗*_, and rearranging to solve for *v*_1D_ (derivation in Supporting Information),
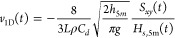
4where *L* is a constant representing the mean distance from the tidally varying shoreline to the 5 m isobath, *h*_5*m*_ is the depth at the 5 m isobath, *g* is gravity, *S*_*xy*_ is the time-varying off-diagonal component of the wave radiation stress tensor, and *H*_*s*,5*m*_ is the significant wave height at the 5 m isobath.

1D model performance depends on the accuracy of eq 4 and the assumption that the grid and *k*_*P*_ are alongshore-uniform (no shoreline curvature, effect of rip currents, and TJRE plume distributed evenly across grid). To test the assumptions of the 1D model method not related to eq 4, eq 1 was also solved with the alongshore-varying nearshore alongshore velocity extracted from the SD Bight model, *v*_C_(*t*,*y*) . This run will be referred to as the “1DC model”, with dye output *C*_1DC_. “1D model” refers to the model run using the alongshore-uniform *v*_1D_(*t*) estimated from eq 4, with dye output *C*_1D_. The grid resolution, time step, and dye loss parameters (*k*_*P*_ and *k*_*B*_) were the same for both runs. The 1DC model can be viewed as an upper-bound of 1D model performance.

Dye was added to the 1D model using a Dirichlet boundary condition, constant *C*_0_ at *y* = 0 km. This boundary condition represents the mean dye concentration adjacent to the PB outfall. The boundary condition *C*_0_ was tuned to parametrize the elevated rate of dye loss near the source and reduced transport efficiency due to alongshore variations (slowdowns and reversals) in nearshore alongshore velocity. The resulting *C*_0_ maximizes 1D model skill in reproducing dye distributions during the tuning period at *y* > 5 km, where the recreational beaches of interest are. The 1DC model used a separately tuned *C*_0_ because it included alongshore variation in nearshore alongshore velocity. When either tuning parameter, *C*_0_ or *C*_*d*_, was varied within an order of magnitude of its optimized value, the impact on the model performance was small.

### Performance Metrics

1D model performance was evaluated by comparing *C*_1D_ and *C*_1DC_ with *C*_C_ (nearshore dye extracted from the SD Bight model) from January 1, 2018, to December 25, 2019 (2 years following the 1 year tuning period). Three performance metrics were used: Pearson’s correlation coefficient (R), the normalized root-mean-square-error (NRMSE), and Willmott’s skill score^42^ (WSS, defined in Supporting Information). To calculate the NRMSE, the root-mean-square error was normalized by the time-averaged value of *C*_C_ for each alongshore location.

The condition *C*_BAC_ = 5 × 10^–4^ was chosen as a cut off value, referred to as the beach advisory condition. This *C*_BAC_ was chosen by converting the dye concentration in the PB outfall (set to 0.7, where 0.01 is 1 part dye to 100 parts water, in the SD Bight model to represent the untreated sewage fraction of the effluent) first to the norovirus abundance in fresh untreated sewage, then to the likelihood of swimmer illness given exposure to the norovirus abundance following Feddersen et al.^16^ Here, *C*_BAC_ corresponds to a 10% likelihood of swimmer illness.^10,16^

Specificity and Sensitivity are calculated,
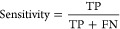
5
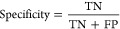
6where Sensitivity describes the True Positives detected out of all possible detectable positives and the Specificity describes the True Negatives detected out of all possible detectable negatives. Both Sensitivity and Specificity range from 0 to 1, with 1 being best.

### Observed Wave Forcing and Water Quality Sampling

The 1D nearshore model was compared with published data from two SADB WTP plume microbial source tracking (MST) sampling campaigns in October 2018 and 2019 by the Southern California Coastal Water Research Project (SCCWRP).^12^ Two of the four sampling campaigns in Zimmer-Faust et al.^12^ were used for comparison because the other two missed the SADB WTP plume or failed to isolate it from the TJRE plume. The samples were tested for three genetic markers, HF183, Lachno3, and *Enterococcus*, with digital droplet PCR (ddPCR). Genetic indicators are an appropriate comparison with model dye because traditional water quality testing by *Enterococcus* culture does not isolate human sources from animal and environmental sources and *Enterococcus* culturability can be impacted by light and salinity.^43^ The nearshore model was forced with advection estimated from historic wave observations furnished by the Coastal Data Information Program, Integrative Oceanography Division, operated by the Scripps Institution of Oceanography (SIO). Wave-driven advection was tuned using nearshore acoustic Doppler current profiler (ADCP) data from Imperial Beach deployed by the Coastal Processes Group at SIO (details in Supporting Information).

## Results and Discussion

### Model Results and Skill

**Figure 2:**
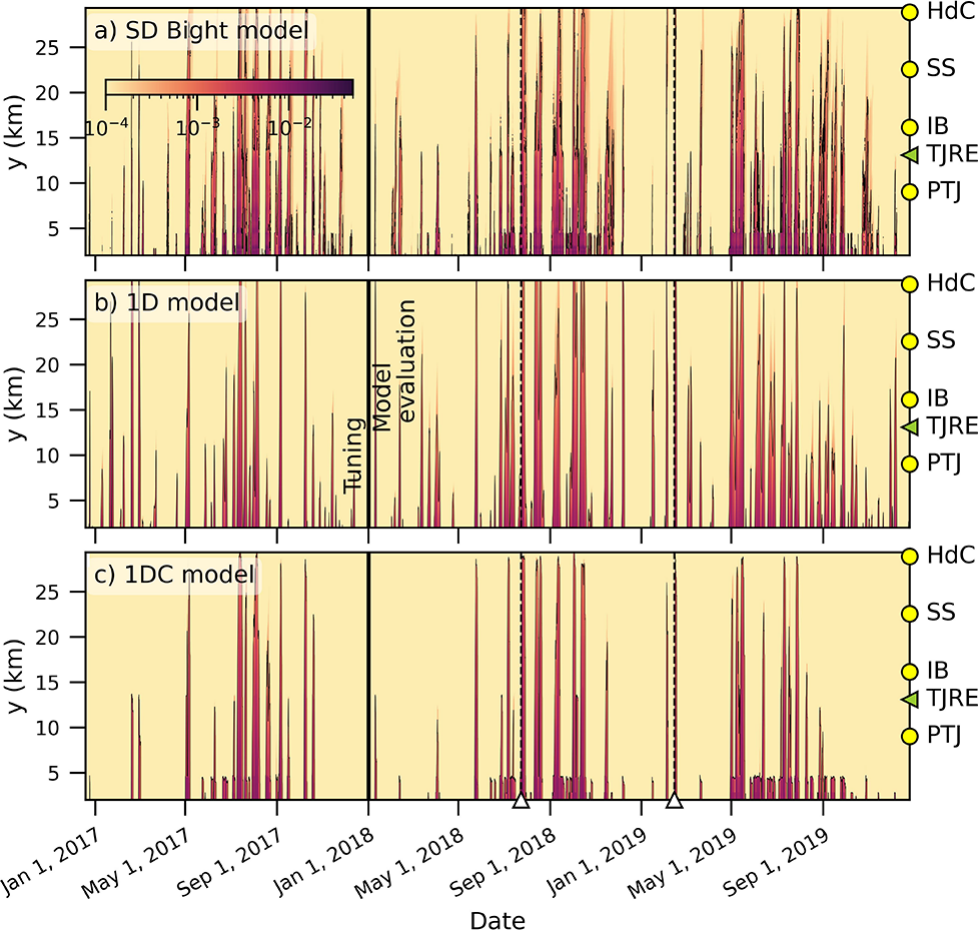
Dye concentration, *C*(*t*, *y*), for *y* > 0 km and for tuning (Dec. 12, 2016, to Dec. 31, 2017) and model performance evaluation periods (Jan. 1, 2018, to Dec. 25, 2019) from the (a) SD Bight model, (b) 1D model, and (c) 1DC model. Arrows on the bottom axis and dashed vertical lines highlight July 24, 2018, and February 14, 2019, the onsets of example summer and winter plume events discussed in the text. Beach locations marked on RHS as in Figure 1. Contour is *C*_BAC_ = 5 × 10^–4^.

1D and 1DC model dyes, *C*_1D_ and *C*_1DC_, respectively, were compared with *C*_C_ for the alongshore region *y* > 0 km over the SD Bight model evaluation period (Figure 2). Seasonal patterns in *C*_C_ were reproduced in *C*_1D_ and *C*_1DC_. More dye was transported northward during summer months (between June 1 and October 1) than nonsummer months in all models. Dye plumes in *C*_C_, *C*_1D_, and *C*_1DC_ reached *y* > 20 km most frequently in the summer (Figure 2a,b). Dye plumes that reached *y* > 20 km during the summer exceeded *C*_BAC_ for many days. An example plume beginning July 24, 2018, exceeded *C*_BAC_ at *y* = 20 km for 5 days in all three models. Winter dye plumes were briefer, on average. A plume that reached *y* > 20 km beginning February 14, 2019, exceeded *C*_BAC_ for just 1 day in the SD Bight model and 2.5 days in the 1D and 1DC models, typical for winter conditions.^44^ More dye was transported northward during summertime because the alongshore nearshore velocity was persistently northward. In winter, northwesterly waves drive predominantly southward nearshore alongshore currents resulting in less dye transport in all models, despite episodic south swells driving nearshore alongshore currents greater than 0.5 m s^–1^ (more on nearshore alongshore velocities in Supporting Information).

**Figure 3:**
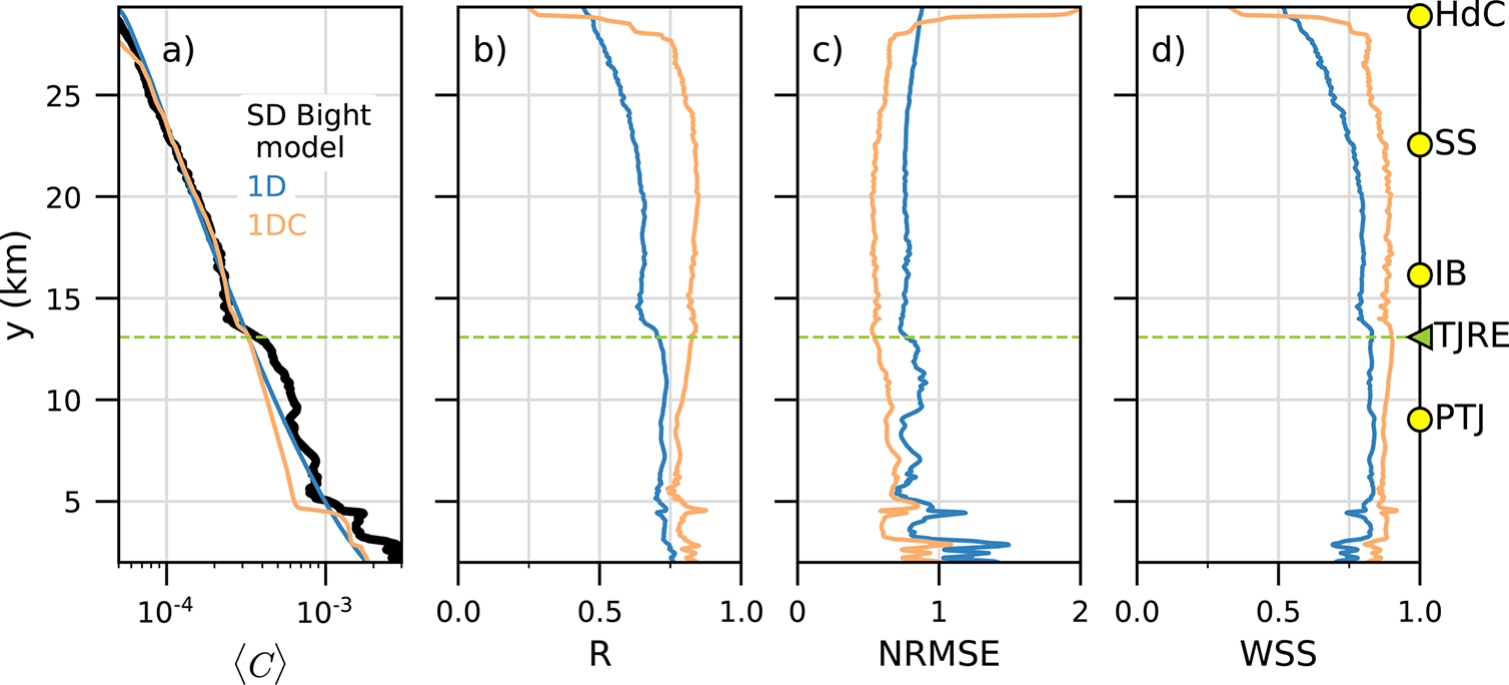
1D (blue) and 1DC (orange) model performance during the model evaluation period as a function of *y*. (a) Time-averaged nearshore dye, 1D, and 1DC overlaid on the SD Bight model (solid black line). Performance metrics are (b) R, (c) NRMSE, and (d) WSS. Green dashed line is the location of TJRE. Beach locations marked on the RHS as in previous figures.

Time-averaged nearshore dye in the 1D and 1DC models, ⟨*C*_1D_⟩ and ⟨*C*_1DC_⟩, respectively, matched the magnitude and alongshore decay of the time-averaged nearshore dye of the SD Bight model, ⟨*C*_C_⟩ (Figure 3). The correlation with ⟨*C*_C_⟩ during the model evaluation period at *y* > 5 km for ⟨*C*_1D_⟩ was *R*^2^ = 0.96 and for ⟨*C*_1DC_⟩ was *R*^2^ = 0.97 (Figure 3a). The three performance metrics (R, NRMSE, and WSS) as a function of *y* statistically quantified the ability of the 1D and 1DC model runs to reproduce nearshore dye from the SD Bight model during the model evaluation period (Figure 3b–d). Since the 1DC model uses the exact alongshore velocities from the SD Bight model, it is expected to act as an upper limit on the 1D model performance. Model skill was high for both, with mean WSS = 0.84 for the 1DC model and 0.75 for the 1D model. 1D model WSS was highest south of TJRE (Figure 3d). Across the TJRE mouth, the 1D model skill decreases by 0.1 in R and 0.05 in WSS (Figure 3b,d). The 1DC model performance did not drop at the TJRE, but remained approximately constant with alongshore distance until *y* = 27 km. 1D model skill metrics gradually decreased beginning around *y* = 20 km, and 1DC model skill metrics sharply decreased north of *y* = 27 km (Figure 3b–d). Alongshore velocity extraction is less precise in the north because of lower grid resolution (at times, nearshore is represented with only one cell) and the growing offset between the angle of the shoreline and the grid.

Here recirculation from the inner shelf was neglected, in contrast with Grimes et al.^7^ who found that differentiating and including recirculation between the surf zone and the inner-shelf (i.e., a 2 box model in the cross-shore direction) increased the performance of a 1D wave-driven advection model. However, the nearshore region used here includes the offshore extent of the inner shelf box from Grimes et al.,^7^ beyond which Grimes et al.^7^ similarly modeled tracer exchange as monotonic decay.

### Binary Performance Using a Cutoff Value

Remaining analyses will use only the 1D model. Recalling that dye represents the concentration of pathogens in untreated wastewater, the *C*_BAC_ threshold represents a dye concentration that would justify issuing a beach advisory. Four conditions are defined using a binary logic criteria of dye greater than *C*_BAC_, taking *C*_C_ as the true result,

1. True Positive: both *C*_1D_ > *C*_BAC_ and *C*_C_ > *C*_BAC_
2. False Positive: *C*_1D_ > *C*_BAC_ but *C*_C_ < *C*_BAC_
3. False Negative: *C*_1D_ < *C*_BAC_ but *C*_C_ > *C*_BAC_
4. True Negative: both *C*_1D_ < *C*_BAC_ and *C*_C_ < *C*_BAC_

A demonstration of the binary criteria at one location is given in Figure S2.

**Figure 4:**
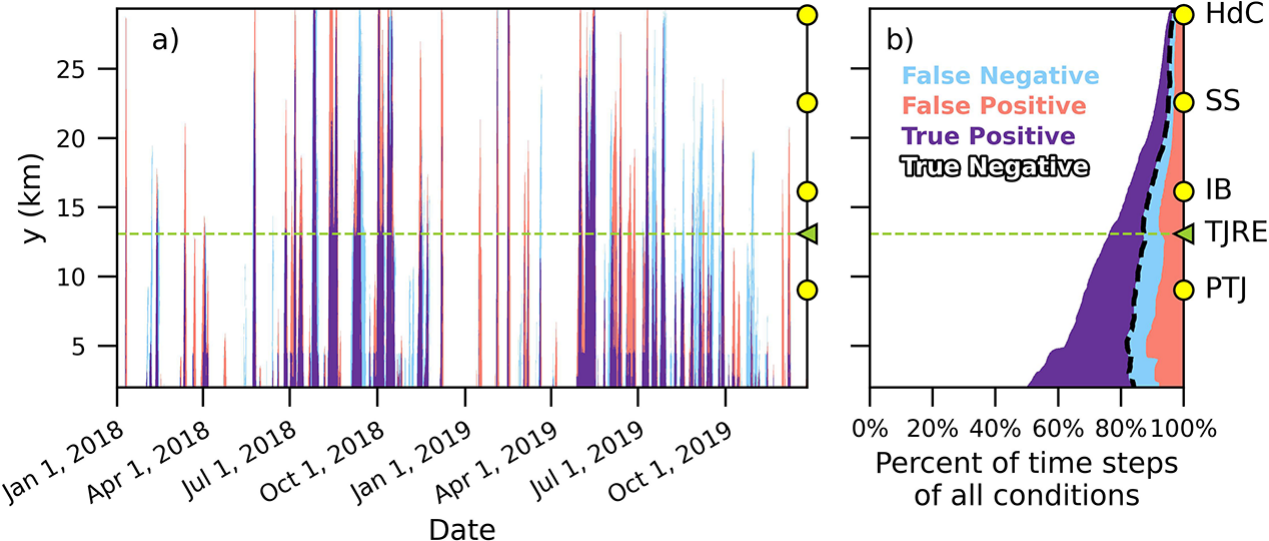
(a) Time series comparing the binary conditions *C*_C_ > *C*_BAC_ and *C*_1D_ > *C*_BAC_ as a function of *y* during the model evaluation period, (b) horizontal stacked bar plot of the percentage of occurrences of the four conditions as a function of *y*. Dashed line is the percent of all time steps in the model evaluation period where models are in agreement, i.e., True Positive or True Negative. Four binary conditions are defined in the text. Example time series at IBP in Figure S2.

This binary analysis was applied to all shoreline locations for all time steps of the model evaluation period (Figure 4a). Agreement was defined as the combined number of hourly time steps that had True Positive or True Negative conditions, and disagreement was defined as either False Positive or False Negative conditions. The 1D model and SD Bight model were in agreement for 89% of time steps at alongshore locations *y* > 0 km (dashed line in Figure 4b). The most common condition was True Negative, accounting for 77% of all hourly time steps at all locations. True Positives were 12%, False Positives were 6%, and False Negatives were 5% (Figure 4b). The percent of time steps in agreement increased with *y* (Figure 4b), in contrast to the pattern in the model skill (Figure 3). This increase is due to an increase in the True Negatives with *y* (Figure 4b). True Negatives accounted for 92% of time steps north of *y* > 20 km because dye concentrations exceeding *C*_BAC_ rarely reached *y* > 20 km in either model.

### Daily Beach Advisories: Comparing 1D Model Forecast with Simulated Weekly Sampling

**Figure 5:**
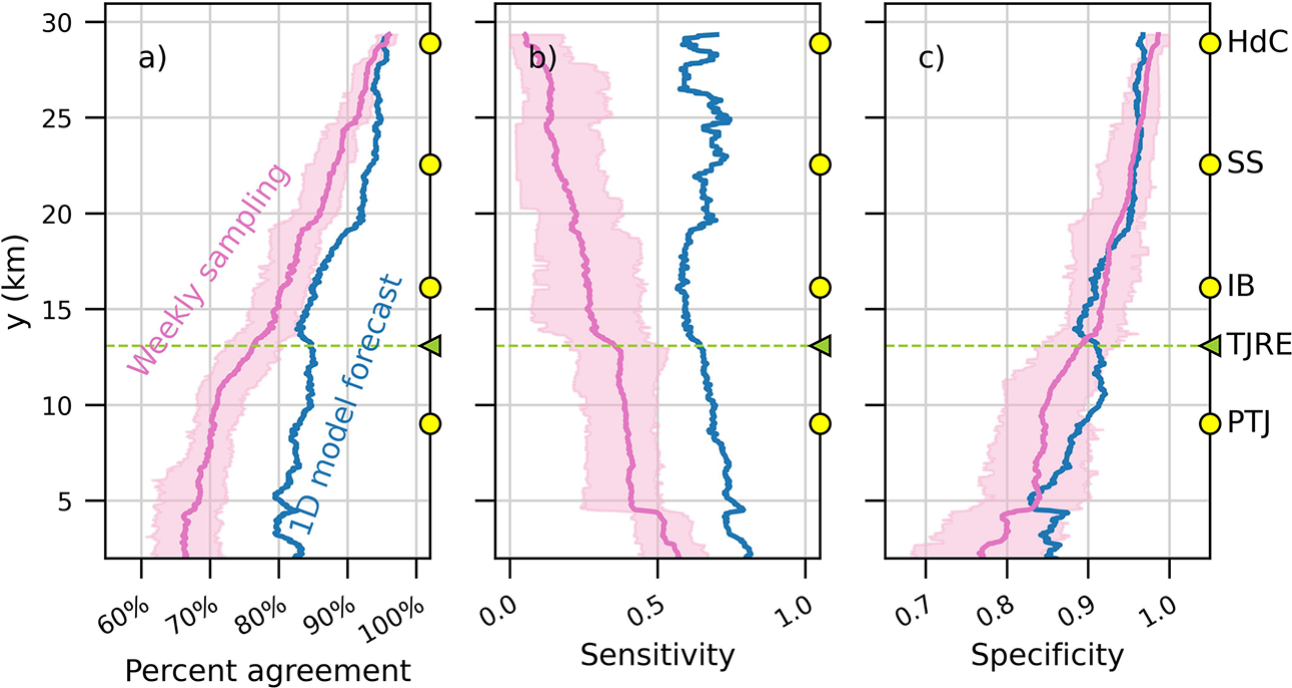
(a) Percent agreement with ideal daily beach advisories (days when *C*_C_ > *C*_BAC_ for any hourly time step from Jan 1, 2018, to Dec 25, 2019) of simulated weekly sampling-informed daily beach advisories (pink) and 1D model-informed daily beach advisories (blue). Simulated weekly sampling was run for every possible sampling hour choice for weekly sampling. Pink line is the mean simulated weekly sampling-informed daily beach advisory accuracy, and pink shading fills from minimum to maximum accuracy. (b) Sensitivity and (c) Specificity of the 1D model forecast compared with simulated weekly sampling.

The previous analysis considered model agreement by hour, but the relevant agreement time scale would be daily, as beach advisories are issued daily. Here we relate the likelihood of an incorrect daily beach advisory posting informed by the 1D model forecast with simulated weekly water quality sampling. A weekly sampling schedule is currently the minimum frequency recommended for water quality monitoring at heavily used urban beaches by the U.S. EPA.^45^ Samples are sent to laboratories for analysis and if FIB are above public health thresholds, beach advisories can be issued the next day.^46^ However, a study on FIB sampling frequency at beaches in Los Angeles, CA, found that a weekly testing schedule missed up to 75% of FIB exceedances, which frequently lasted only 1 day.^47^ Although here dye was modeled with the 10 day decay rate of norovirus and not FIB, weekly sampling is still likely to misrepresent dye presence because dye concentrations were determined primarily by alongshore advection, which acted on shorter time scales. For this experiment, the ideal daily beach advisory was issued at an alongshore location if *C*_C_ > *C*_BAC_ for at least 1 h during that day. A 1D model-informed daily beach advisory was issued at an alongshore location if *C*_1D_ > *C*_BAC_ for at least 1 h during that day. To simulate weekly sampling, *C*_C_ was checked at one time step once per week (the “sampling hour”). Simulations were run for each possible sampling hour, presenting a range of results. If the weekly sample exceeded *C*_BAC_, a daily beach advisory was issued the following day (to match the time lag required to culture samples) and remained in place for the next 7 days until the next sample was processed. Accuracy was determined by checking if the 1D model-informed and simulated weekly sampling-informed daily beach advisories matched the ideal beach advisory. The magnitude and shape of the curve for 1D model-informed daily beach advisory accuracy (Figure 5) were consistent with the hourly agreement between binary metrics *C*_C_ > *C*_BAC_ and *C*_1D_ > *C*_BAC_ (Figure 4b). The accuracy of simulated weekly sampling-informed daily beach advisories varied with the sampling hour choice. The difference between mean accuracy (pink line in Figure 5) and minimum or maximum accuracy (pink shading in Figure 5) could be up to ±9%, but had an alongshore-average of ±3.5%. The mean accuracy varied alongshore from 65% to 96% with an average of 78%, consistent with the range of 0–40% inaccuracy in weekly sampling-informed daily beach advisories estimated for Huntington Beach, CA.^48^ The 1D model-informed daily beach advisories varied in accuracy from 79% to 96% with an average of 87%. The 1D model-informed beach advisories were more accurate than mean simulated weekly sampling by 0–18%, with an alongshore-average improvement of 8% (difference between pink and blue lines in Figure 5a). Similar to hourly agreement, accuracy for both 1D model-informed and simulated weekly sampling-informed daily beach advisories increased with *y* as true negatives increased. Specificity and Sensitivity were calculated (values in Table S2). 1D model-informed daily beach advisories had significantly higher Sensitivity, with a alongshore-mean increase of 0.38 over simulated weekly sampling (Figure 5b). However, the difference in 1D model-informed daily beach advisory Specificity was not significant, with an alongshore-mean increase of just 0.01 over simulated weekly sampling (Figure 5c). Therefore, while much of the 1D model accuracy was attributable to correctly modeling plume absence, the potential improvement over weekly sampling was in detecting plume presence. This suggests that the 1D model offers asignificant improvement over weekly sampling in properly identifying poor water quality events.

### Comparison with Water Sampling

**Figure 6:**
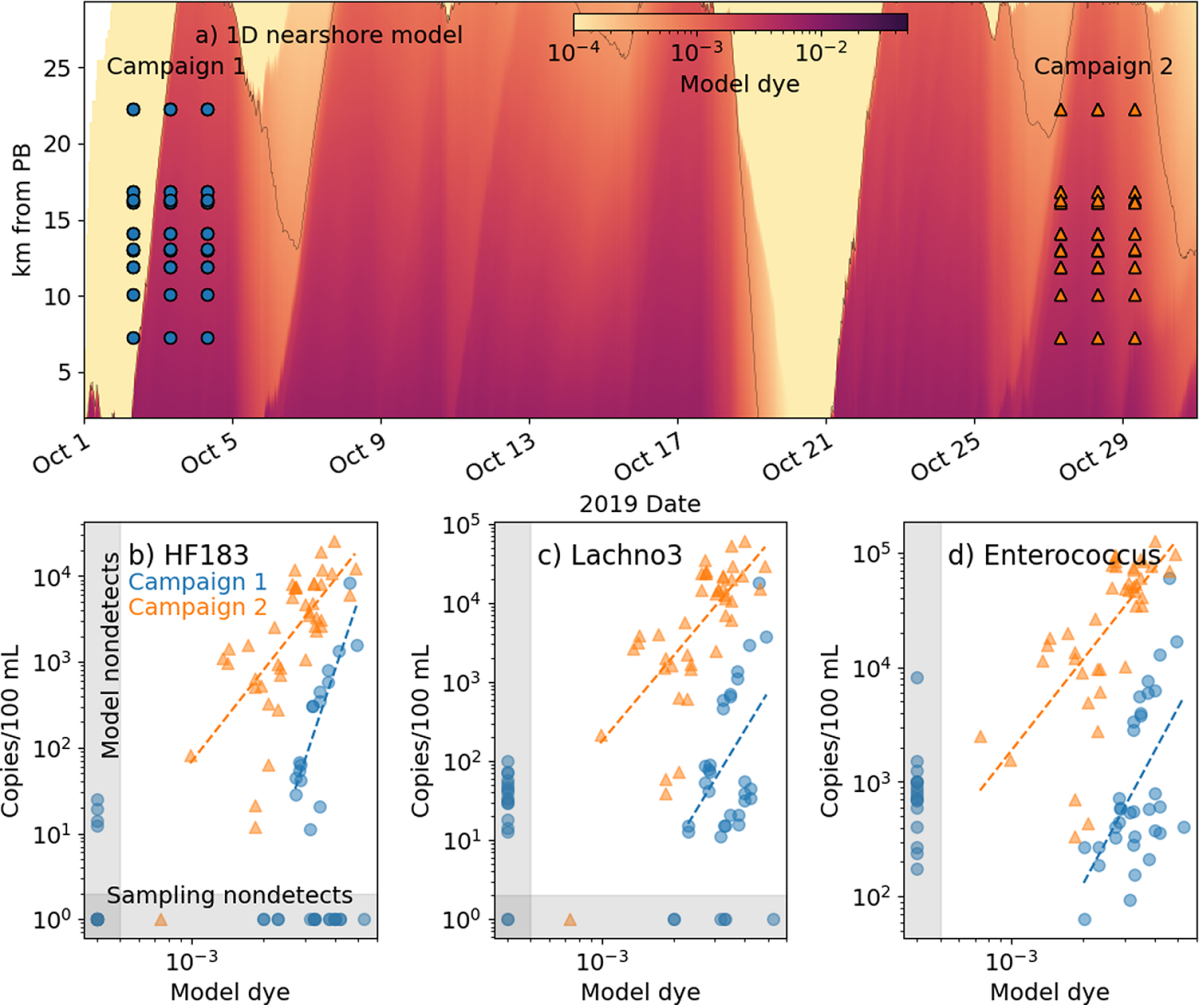
(a) Nearshore model run with historic wave forcing from October 2019 (color, contour is *C*_BAC_ = 5 × 10^–4^) by time and *y*. Circles and triangles indicate times and locations of two MST sampling campaigns from Zimmer-Faust et al.^12^ (blue circles = Campaign 1 on Oct. 2–4, orange triangles = Campaign 2 on Oct. 27–29). MST indicators were human-associated genetic markers HF183 and Lachno3, and genetically sampled (not cultured) *Enterococcus*. (b–d) Log model dye against log MST indicator copies color-coded by sampling campaign. Slopes, intercepts, and correlations of linear fits for each indicator and sampling campaign are listed in Table S3.

The 1D model was run with historical wave forcing observed during two water quality sampling campaigns targeting the SABWTP plume using ddPCR (Figure 6). 1D model dye was linearly fit in log–log space to observed DNA copies of three genetic markers: HF183, Lachno3, and *Enterococcus*. Best fit line slopes differed more between sampling campaigns than across genetic markers (Table S3), as in previous literature.^49^ The combined physical and biological loss rate of all genetic markers was faster by an average of 74% during October 2–4 sampling than October 27–29, suggesting temporal variability in either offshore export (because transient rip currents vary with incoming wave directional spread) or indicator decay (because decay varies with temperature). The effects of offshore export and genetic marker decay cannot be uncoupled in the observations, precluding a comparison of estimated loss rates with genetic marker decay rates in the literature. Sensitivity and Specificity were evaluated by counting nondetects below the BAC threshold for the 1D model and 1 copy/100 mL for samples (Table S3). High Sensitivities and low Specificities were found for all genetic markers (Table S3). Low Specificity may be due to few nondetects with targeted plume sampling.

### Effect of Tijuana River Estuary on Model Performance

1D model performance was lower downstream of TJRE (Figure 3). This decreased performance may be attributed to decreases or reversals in the alongshore velocity at TJRE, because the performance did not decrease in the 1DC model. However, this decrease in 1D model performance at TJRE was not found in the binary analysis, which only considered dye exceeding *C*_BAC_. In the binary analysis, the agreement between the 1D model and the SD Bight model increased with *y* (Figure 4). This suggests the effect of the TJRE may primarily concern lower dye concentrations, and alongshore variations in velocity at TJRE may be negligible for beach management concerns in this region. However, future work is needed to explicitly explore the dynamical role of inlets and shoals on nearshore alongshore tracer transport, as these are common shoreline features. Estuary mouths along different shorelines may have larger effects.

### Effect of Neglecting Nonwave Forcing in Alongshore Momentum

Only wave-driven velocities were included in estimating *v*_1D_ for the 1D model to optimize model performance with simplicity. Successful comparison with the 1DC, which uses the hydrodynamic model alongshore velocity with all alongshore momentum terms, justifies the neglect of nonwave forcing for calculating *v*_1D_. The second leading-order term in the nearshore alongshore momentum balance has been observed to be wind stress.^18,19^ The improvement in model performance by including wind here is likely to be small since in this region fair weather winds were light and strong winds accompanied rainfall, already a recognized condition for posting beach advisories. However, wind stress may be more important for nearshore transport during wet weather or in other regions. For example, in Melbourne Beach, FL, where hurricanes are common, the correlation of wind stress with waves explained net sediment transport better than waves alone.^50^

### Applications for Beach Management

Here tracer was modeled on norovirus because quantitative microbial risk assessment has found norovirus to be the greatest contributor to GI illness likelihood in marine swimmers,^51,52^ but EPA guidelines use cultured *Enterococcus* as the basis for beach advisories for marine waters.^3^ However, this model could be used as a conservative forecast of cultured during dry weather even without modification to tracer behavior. The model could be used to optimize sampling effort by targeting FIB sampling at beaches that are forecast to be likely exposed to coastal pollution. If available FIB sampling methods cannot return same day results, a temporary swim-at-your-own-risk posting could explain why marine pollution is likely to be present (based on known pollution sources and wave swell direction) and that a final advisory would be posted when sampling results were returned. Model-informed dry weather sampling advisories would complement the existing strategy of posting beach advisories after rainfall.

Modeled tracer could be made more like traditionally cultured *Enterococcus* by programming UV-dependent decay.^33,53,54^ Note however that tuning and validation of biological model improvements would be limited by the performance of the physical transport model.

### Applications for Other Regions and Tracers

The 1D wave-driven advection model tested here could be adapted to model the nearshore transport of other tracers on other mostly straight, wave-dominated coastlines using wave buoys or wave models where data are available to tune *k*_*P*_ and *C*_*d*_. Tuning *C*_*d*_ could be done with validated hydrodynamic models or with nearshore velocity measurements and *k*_*P*_ could be tuned using measured or inferred alongshore loss of the specific tracer or a proxy. Although a uniform value of *k*_*P*_ was used here, alongshore variation may be necessary for other coastlines. Wind stress may be added to the alongshore momentum equation where appropriate. For example, a similar 1D model could predict the wave-driven nearshore transport of microplastics.^55^ Although here we used a persistent flux of polluted waters, a time-dependent source term could represent transient sources of pollution to wave-dominated coastlines. For example, FIB levels are elevated in rivers in the days following hurricanes in North Carolina.^56,57^ Those polluted rivers form buoyant plumes at the coast which are partially trapped in the nearshore,^58,59^ and a similar 1D model could be used to model the wave- and wind-driven fate of those plumes along the shoreline. Because the 1D model is simple, it could be coupled to models that currently use only shelf circulation. Offline particle tracking algorithms used to model transport of harmful algal blooms^60^ or larvae^61^ using shelf currents could implement a nested 1D nearshore wave-driven transport model. Bathymetric nonuniformities should be considered in models at new sites. Although here model performance was good while neglecting bathymetric nonuniformities, this result may not be generalizeable.

## Supporting Information Available

Figures S1 and S2, Tables S1–S3 and additional methods, results, and analysis (PDF)

## References

[ref1] Shuval H. Estimating the global burden of thalassogenic diseases: human infectious diseases caused by wastewater pollution of the marine environment. Journal of Water and Health 2003, 1, 53–64. 10.2166/wh.2003.0007. 15382734

[ref2] de Brauwere A.; Ouattara N. K.; Servais P. Modeling Fecal Indicator Bacteria in Natural Surface Waters: A Review. Critical Reviews in Environmental Science and Technology 2014, 44, 2380–2453. 10.1080/10643389.2013.829978.

[ref3] Arcadis. Tijuana River Diversion Study; 2019. https://www.nadb.org/uploads/files/tijuana_river_diversion_study_final_report_full_sm.pdf (accessed 2023-09-07).

[ref4] Grant S. B.; Kim J. H.; Jones B. H.; Jenkins S. A.; Wasyl J.; Cudaback C. Surf zone entrainment, along-shore transport, and human health implications of pollution from tidal outlets. J. Geophys. Res. 2005, 110, C10025 10.1029/2004JC002401.

[ref5] Hally-Rosendahl K.; Feddersen F.; Clark D. B.; Guza R. T. Surfzone to inner-shelf exchange estimated from dye tracer balances. Journal of Geophysical Research: Oceans 2015, 120, 6289–6308. 10.1002/2015JC010844.

[ref6] Feddersen F.; Olabarrieta M.; Guza R. T.; Winters D.; Raubenheimer B.; Elgar S. Observations and Modeling of a Tidal Inlet Dye Tracer Plume. Journal of Geophysical Research: Oceans 2016, 121, 7819–7844. 10.1002/2016JC011922.

[ref7] Grimes D. J.; Feddersen F.; Giddings S. N. Long-distance/time surf-zone tracer evolution affected by inner-shelf tracer retention and recirculation. J. Geophysical Research Oceans 2021, 126, e2021JC017661 10.1029/2021JC017661.

[ref8] San Diego County San Diego Beach Information. http://www.sdbeachinfo.com/ (accessed 2021-06-14).

[ref9] Boehm A. B.; Ashbolt N. J.; Colford J. M.; Dunbar L. E.; Fleming L. E.; Gold M. A.; Hansel J. A.; Hunter P. R.; Ichida A. M.; McGee C. D.; Soller J. A.; Weisberg S. B. A sea change ahead for recreational water quality criteria. Journal of Water and Health 2009, 7, 9–20. 10.2166/wh.2009.122. 18957771

[ref10] Boehm A.; Soller J. A. Refined ambient water quality thresholds for human-associated fecal indicator HF183 for recreational waters with and without co-occurring gull fecal contamination. Microbial Risk Analysis 2020, 16, 100139 10.1016/j.mran.2020.100139.

[ref11] Rippy M. A.; Stein R.; Sanders B. F.; Davis K.; McLaughlin K.; Skinner J. F.; Kappeler J.; Grant S. B. Grant, S. B. Small Drains, Big Problems: The Impact of Dry Weather Runoff on Shoreline Water Quality at Enclosed Beaches. Environ. Sci. Technol. 2014, 48, 14168–14177. 10.1021/es503139h. 25390647

[ref12] Zimmer-Faust A. G.; Steele J. A.; Xiong X.; Staley C.; Griffith M.; Sadowsky M. J.; Diaz M.; Griffith J. F. A combined digital PCR and next generation DNA-sequencing based approach for tracking nearshore pollutant dynamics along the southwest U.S/Mexico border. Front. Microbiol. 2021, 12, 674214 10.3389/fmicb.2021.674214. 34421839 PMC8377738

[ref13] Kim S. Y.; Terrill E. J.; Cornuelle B. D. Assessing coastal plumes in a region of multiple discharges: The US-Mexico border. Environ. Sci. Technol. 2009, 43, 7450–7457. 10.1021/es900775p. 19848160

[ref14] Wu X.; Feddersen F.; Giddings S. N.; Kumar N.; Gopalakrishnan G. Mechanisms of Mid- to Outer-Shelf Transport of Shoreline-Released Tracers. Journal of Physical Oceanography 2020, 50, 1813–1837. 10.1175/JPO-D-19-0225.1.

[ref15] Rogowski P. A.; Terrill E.; Schiff K.; Kim S. Y. An assessment of the transport of southern California stormwater ocean discharges. Mar. Pollut. Bull. 2015, 90, 135–142. 10.1016/j.marpolbul.2014.11.004. 25467870

[ref16] Feddersen F.; Boehm A. B.; Giddings S. N.; Wu X.; Liden D. Modeling untreated wastewater evolution and swimmer illness for four wastewater infrastructure scenarios in the San Diego-Tijuana (US/MX) border region. GeoHealth 2021, 5, e2021GH000490 10.1029/2021GH000490. PMC8581746 34796313

[ref17] Thornton E. B.; Guza R. T. Surf zone longshore currents and randome waves: Field data and models. J. Phys. Ocean. 1986, 16, 1165–1178. 10.1175/1520-0485(1986)016<1165:SZLCAR>2.0.CO;2.

[ref18] Feddersen F. Weakly nonlinear shear waves. J. Fluid Mech. 1998, 372, 71–91. 10.1017/S0022112098002158.

[ref19] Lentz S.; Guza R. T.; Elgar S.; Feddersen F.; Herbers T. H. C. Momentum balances on the North Carolina inner shelf. J. Geophys. Res. 1999, 104, 18205–18240. 10.1029/1999JC900101.

[ref20] Boehm A. Model of Microbial Transport and Inactivation in the Surf Zone and Application to Field Measurements of Total Coliform in Northern Orange County, California. Environ. Sci. Technol. 2003, 37, 5511–5517. 10.1021/es034321x. 14717158

[ref21] Boehm A. B.; Keymer D. P.; Shellenbarger G. G. An analytical model of enterococci inactivation, grazing, and transport in the surf zone of a marine beach. Water Res. 2005, 39, 3565–3578. 10.1016/j.watres.2005.06.026. 16095656

[ref22] Elko N.; Foster D.; Kleinheinz G.; Rubenheimer B.; Brander S.; Kinzelman J.; Kritzer J.; Monroe D.; Storlazzi C.; Sutula M.; Mercer A.; Coffin S.; Fraioli C.; Ginger L.; Morrison E.; Parent-Doliner G.; Akan C.; Canestrelli A.; DiBenedetto M.; Lang J.; Simm J. Coastal Forum: Human and ecosystem health in coastal systems. Shore and Beach 2022, 90, 64–91.

[ref23] Wu X.; Feddersen F.; Giddings S. N. Automated temporal tracking of coherently evolving density fronts in numerical models. Journal of Atmospheric and Oceanic Technology 2021, 38, 2095–2108. 10.1175/JTECH-D-21-0072.1.

[ref24] Wu X.; Feddersen F.; Giddings S. N. Characteristics and Dynamics of Density Fronts over the Inner to Mid-shelf under Weak Wind Conditions. Journal of Physical Oceanography 2021, 51, 789–808. 10.1175/JPO-D-20-0162.1.

[ref25] Wu X.; Feddersen F.; Giddings S. N. Diagnosing surfzone impacts on inner-shelf flow spatial variability using realistic model experiments with and without surface gravity waves. Journal of Physical Oceanography 2021, 2505–2515. 10.1175/JPO-D-20-0324.1.

[ref26] Kumar N.; Voulgaris G.; Warner J. C.; Olabarrieta M. Implementation of the vortex force formalism in the coupled ocean-atmosphere-wave-sediment transport (COAWST) modeling system for inner shelf and surf zone applications. Ocean Modelling 2012, 47, 65–95. 10.1016/j.ocemod.2012.01.003.

[ref27] Warner J. C.; Armstrong B.; He R.; Zambon J. B. Development of a Coupled Ocean-Atmosphere-Wave-Sediment Transport (COAWST) Modeling System. Ocean Modelling 2010, 35, 230–244. 10.1016/j.ocemod.2010.07.010.

[ref28] Shchepetkin A. F.; McWilliams J. C. The regional oceanic modeling system (ROMS): a split-explicit, free-surface, topography-following-coordinate oceanic model. Ocean Modelling 2005, 9, 347–404. 10.1016/j.ocemod.2004.08.002.

[ref29] Booij N.; Ris R. C.; Holthuijsen L. H. A third-generation wave model for coastal regions: 1. Model description and validation. Journal of Geophysical Research: Oceans 1999, 104, 7649–7666. 10.1029/98JC02622.

[ref30] Steets B. M.; Holden P. A. A mechanistic model of runoff-associated fecal coliform fate and transport through a coastal lagoon. Water Res. 2003, 37, 589–608. 10.1016/S0043-1354(02)00312-3. 12688694

[ref31] Jamieson R.; Joy D. M.; Lee H.; Kostaschuk R.; Gordon R. Transport and deposition of sediment-associated Escherichia coli in natural streams. Water Res. 2005, 39, 2665–2675. 10.1016/j.watres.2005.04.040. 15979685

[ref32] Cho K. H.; Cha S. M.; Kang J. H.; Lee S. W.; Park Y.; Kim J. W.; Kim J. H. Meteorological effects on the levels of fecal indicator bacteria in an urban stream: A modeling approach. Water Res. 2010, 44, 2189–2202. 10.1016/j.watres.2009.12.051. 20138642

[ref33] Boehm A. B.; Graham K. E.; Jennings W. C. Can We Swim Yet? Systematic Review, Meta-Analysis, and Risk Assessment of Aging Sewage in Surface Waters. Environ. Sci. Technol. 2018, 52, 9634–9645. 10.1021/acs.est.8b01948. 30080397

[ref34] Hally-Rosendahl K.; Feddersen F.; Guza R. T. Cross-shore tracer exchange between the surfzone and inner-shelf. Journal of Geophysical Research: Oceans 2014, 119, 4367–4388. 10.1002/2013JC009722.

[ref35] Moulton M.; Elgar S.; Raubenheimer B.; Warner J. C.; Kumar N. Rip currents and alongshore flows in single channels dredged in the surf zone. Journal of Geophysical Research: Oceans 2017, 122, 3799–3816. 10.1002/2016JC012222.

[ref36] Moulton M.; Chickadel C.; Thomson J. Warm and Cool Nearshore Plumes Connecting the Surf Zone to the Inner Shelf. Geophys. Res. Lett. 2021, 48, e2020GL091675 10.1029/2020GL091675.

[ref37] Suanda S. H.; Feddersen F. A self-similar scaling for cross-shelf exchange driven by transient rip currents. Geophys. Res. Lett. 2015, 42, 5427–5434. 10.1002/2015GL063944.

[ref38] Kumar N.; Feddersen F. A new offshore transport mechanism for shoreline-released tracer induced by transient rip currents and stratification. Geophys. Res. Lett. 2017, 44, 2843–2851. 10.1002/2017GL072611.

[ref39] Longuet-Higgins M. S. Longshore currents generated by obliquely incident sea waves, parts 1 and 2. J. Geophys. Res. 1970, 75, 6778–6801. 10.1029/JC075i033p06778.

[ref40] Thornton E. B.; Guza R. T. Transformation of wave height distribution. J. Geophys. Res. 1983, 88, 5925–5938. 10.1029/JC088iC10p05925.

[ref41] Wright D. G.; Thompson K. R. Time-averaged forms of the nonlinear stress law. Journal of Physical Oceanography 1983, 13, 341–346. 10.1175/1520-0485(1983)013<0341:TAFOTN>2.0.CO;2.

[ref42] Willmott C. J. On the validation of models. Physical Geography 1981, 2, 184–194. 10.1080/02723646.1981.10642213.

[ref43] Gin K. Y.; Goh S. G. Modeling the effect of light and salinity on viable but non-culturable (VBNC) Enterococcus. Water Res. 2013, 47, 3315–3328. 10.1016/j.watres.2013.03.021. 23602617

[ref44] Ludka B. C.; Guza R. T.; O’Reilly W. C.; Merrifield M. A.; Flick R. E.; Bak A. S.; Hesser T.; Bucciarelli R.; Olfe C.; Woodward B.; Boyd W.; Smith K.; Okihiro M.; Grenzeback R.; Parry L.; Boyd G. Sixteen years of bathymetry and waves at San Diego beaches. Scientific Data 2019, 6, 161 10.1038/s41597-019-0167-6. 31467271 PMC6715754

[ref45] U.S. Environmental Protection Agency. National Beach Guidance Criteria and Required Performance for Grants; 2014.

[ref46] Francy D. Use of predictive models and rapid methods to nowcast bacteria levels at coastal beaches. Aquatic Ecosystem Health and Management 2009, 12, 177–182. 10.1080/14634980902905767.

[ref47] Leecaster M. K.; Weisberg S. B. Effect of Sampling Frequency on Shoreline Microbiology Assessments. Mar. Pollut. Bull. 2001, 42, 1150–1154. 10.1016/S0025-326X(01)00130-8. 11763228

[ref48] Kim J. H.; Grant S. B. Public Mis-Notification of Coastal Water Quality: A Probabilistic Evaluation of Posting Errors at Huntington Beach, California. Environ. Sci. Technol. 2004, 38, 2497–2504. 10.1021/es034382v. 15180043

[ref49] Mattioli M. C.; Sassoubre L. M.; Russell T. L.; Boehm A. B. Decay of sewage-sourced microbial source tracking markers and fecal indicator bacteria in marine waters. Water Res. 2017, 108, 106–104. 10.1016/j.watres.2016.10.066. 27855952

[ref50] Burnette C.; Dally W. R. The Longshore Transport Enigma and Analysis of a 10-Year Record of Wind-driven Nearshore Currents. Journal of Coastal Research 2018, 341, 26–41. 10.2112/JCOASTRES-D-17-00010.1.

[ref51] Soller J. A.; Bartrand T.; Ashbolt N. J.; Ravenscroft J.; Wade T. J. Estimating the primary etiologic agents in recreational freshwaters impacted by human sources of faecal contamination. Water Res. 2010, 44, 4736–4747. 10.1016/j.watres.2010.07.064. 20728915

[ref52] Boehm A. B.; Soller J. A.; Shanks O. C. Human-Associated Fecal Quantitative Polymerase Chain Reaction Measurements and Simulated Risk of Gastrointestinal Illness in Recreational Waters Contaminated with Raw Sewage. Environmental Science & Technology Letters 2015, 2, 270–275. 10.1021/acs.estlett.5b00219.

[ref53] Davies-Colley R. J.; Bell R. G.; Donnison A. Sunlight inactivation of enterococci and fecal coliforms in sewage effluent diluted in seawater. Appl. Environ. Microbiol. 1994, 60, 2049–2058. 10.1128/aem.60.6.2049-2058.1994. 16349290 PMC201600

[ref54] Byappanahalli M. N.; Nevers M. B.; Korajkic A.; Staley Z. R.; Harwood V. J. Enterococci in the environment. Microbiology and Molecular Biology Reviews 2012, 76, 685–706. 10.1128/MMBR.00023-12. 23204362 PMC3510518

[ref55] Kerpen N. B.; Schlurmann T.; Schendel A.; Gundlach J.; Marquard D.; Hupgen M. Wave-Induced Distribution of Microplastics in the Surf Zone. Frontiers in Marine Science 2020, 7, 590565 10.3389/fmars.2020.590565.

[ref56] Humphrey C.; Iverson G.; Skibiel C.; Sanderford C.; Blackmon J. Geochemistry of Flood Waters from the Tar River, North Carolina Associated with Hurricane Matthew. Resources 2019, 8, 48 10.3390/resources8010048.

[ref57] Neville J. A.; Emanuel R. E.; Nichols E. G.; Vose J. Extreme Flooding and Nitrogen Dynamics of a Blackwater River. Water Resour. Res. 2021, 57, e2020WR029106 10.1029/2020WR029106.

[ref58] Rodriguez A. R.; Giddings S. N.; Kumar N. Impacts of Nearshore Wave-Current Interaction on Transport and Mixing of Small-Scale Buoyant Plumes. Geophys. Res. Lett. 2018, 45, 8379–8389. 10.1029/2018GL078328.

[ref59] Kastner S. E.; Horner-Devine A. R.; Thomson J. M. A Conceptual Model of a River Plume in the Surf Zone. Journal of Geophysical Research: Oceans 2019, 124, 8060–8078. 10.1029/2019JC015510.

[ref60] Giddings S. N.; MacCready P. M.; Hickey B. M.; Banas N. S.; Davis K. A.; Siedlecki S. A.; Trainer V. L.; Kudela R. M.; Pelland N. A.; Connolly T. P. Hindcasts of potential harmful algal bloom transport pathways on the Pacific Northwest coast. Journal of Geophysical Research-Oceans 2014, 119, 2439–2461. 10.1002/2013JC009622.

[ref61] Brasseale E.; Grason E.; McDonald P. S.; Adams J.; MacCready P. Larval transport modeling support for identifying population sources of European Green Crab in the Salish Sea. Estuaries and Coasts 2019, 42, 1586–1599. 10.1007/s12237-019-00586-2.

