# Supplementary material for: Performance of a One-Dimensional Model of Wave-Driven Nearshore Alongshore Tracer Transport and Decay with Applications for Dry Weather Coastal Pollution
Source: Environ Sci Technol. 2023 Sep 22;57(39):14674–83. doi: 10.1021/acs.est.2c08656 (PMC10552543; doi:10.1021/acs.est.2c08656)
Supplement: Supplementary file 1 — es2c08656_si_001.pdf [file es2c08656_si_001.pdf]

Supporting Information for: Performance of a one-dimensional model of wave-driven nearshore alongshore tracer transport and decay with applications for dry weather coastal pollution

Elizabeth Brasseale,[*,†] Falk Feddersen,[†] Xiaodong Wu,[‡] Amity G. Zimmer-Faust,[¶,§] and Sarah N. Giddings[†]

†*Scripps Institution of Oceanography, 9500 Gilman Dr., La Jolla, CA 92093*

‡*School of Oceanography, Shanghai Jiao Tong University, 1954 Huashan Rd., Shanghai 200030, China*

¶*The Nature Conservancy, 830 S Street, Sacramento, CA 96811*

§*Southern California Coastal Water Research Project, 3535 Harbor Blvd Suite 110, Costa Mesa, CA*

E-mail: eabrasse@uw.edu

Number of pages: 12

Number of figures: 2

Number of tables: 3

Supporting Information Available

Determining dye parameters k_P and C_0

A value for k_P was used for all 1D model runs based on dye concentrations from the SD Bight model, and C_0 was tuned using an iterative search optimization method to maximize model performance for each 1D model run. The physical rate of dye loss was determined from $k_P = k - k_B$, where k is the total temporal rate of dye loss from the nearshore region of the SD Bight model. The total temporal rate of dye loss, k, assumed constant in time and space, was estimated using the decrease with y in time-averaged nearshore dye, $\langle C_\mathrm{C} \rangle$. This decrease was exponential in $\langle C_\mathrm{C} \rangle$ north of $y = 5$ km, or equivalently, linear in $\ln\langle C_\mathrm{C} \rangle$. The total temporal decay rate, k, was related to the time-averaged spatial decay rate using a velocity scale, V,

$$k = V \frac{d \ln\langle C_\mathrm{C} \rangle}{dy}. \tag{S1}$$

The velocity scale was chosen to be $V = 0.1\mathrm{ms}^{-1}$, the RMS of $\bar{v}_\mathrm{C}$ (Fig. S1). The mean of $\bar{v}_\mathrm{C}$ was not used because $\bar{v}_\mathrm{C}$ values occurred roughly symmetric around 0 (Fig. S1), such that the mean of $\bar{v}_\mathrm{C}$ was less than the typical velocity magnitude.

Beginning 5 km north of PB, the time-averaged SD Bight dye, $\langle C_\mathrm{C} \rangle$, decays exponentially with y (Fig. 3a). The slope of $\ln\langle C_\mathrm{C} \rangle$ from the tuning period indicates an e-folding length scale of 7.9 km. This length scale was used to derive $k_P = 1.3 \times 10^{-5}$ s^{-1}, an order of magnitude greater than k_B and slightly lower than the estimate of 5×10^{-5} determined for the region between the 4-m isobath and the surf zone edge in Grimes et al.[1]. The optimal Dirichlet boundary conditions for the 1D models were found to be $C_0 = 0.008$ for the 1D model and $C_0 = 0.011$ for the 1DC model. With these k_P and C_0, the 1D and 1DC models were able to reproduce time-averaged dye at alongshore locations with considerable skill.

Calculating velocity from wave properties

The 1D model alongshore-uniform wave-driven nearshore alongshore velocity, $v_{\mathrm{1D}}(t)$, was estimated from wave properties at an offshore location (32.56957 N, -117.1688 E, 20-m isobath, Fig. 1), the position of the Imperial Beach Nearshore Buoy operated by the Coastal Data Information Program (CDIP). To make use of the established relationship between surf zone alongshore currents and waves, (2),[2–4] the alongshore currents in the nearshore region are presumed to be proportional to surf zone alongshore-mean alongshore currents.

To estimate v_{1D}, first the right hand side of (2) was simplified using a finite difference approximation. Radiation stress begins decreasing in the surf zone where waves break, and S_{xy} decreases to zero at the shoreline. To average this wave forcing across the nearshore domain, the change in S_{xy} to zero is divided by the cross-shore distance to the 5-m isobath L,

$$\frac{\partial S_{xy}}{\partial x} \approx \frac{S_{xy}(t)}{L}. \tag{S2}$$

For simplicity and generalizabilty to locations without well-known bathymetry, (S2) was evaluated with a constant L, set to the mean of the tidally-varying distance to the 5-m isobath. A narrow-banded representation of S_{xy} is used,[2]

$$S_{xy}(t) = E(t)\frac{c_g(t)}{c_p(t)}\cos\theta'(t)\sin\theta'(t), \tag{S3}$$

where E is the wave energy, c_g is the group velocity, c_p is the phase velocity, and θ' is the difference between the mean wave direction, θ, from shorenormal, θ_{SN}. For these estimates of alongshore-uniform wave-driven alongshore velocity, θ_{SN} was a constant chosen to optimize model performance. The 1D model was sensitive to the choice of θ_{SN} because wave direction is often near shorenormal, and the sign of θ' determines the direction of the velocity. Over the stretch of shoreline of interest, the mean shorenormal angle is 260°, varying from 240° to 270°. Shorenormal angles are closest to 270° in center and decrease towards the domain edges. Using uniform shorenormal angle $\theta_{\mathrm{SN}} = 263°$ resulted in best R, NRMSE, and WSS

of $v_{1\mathrm{D}}$ out of one hundred θ_{SN} values tested in the range 240° to 270°. The SD Bight model alongshore-varying nearshore alongshore velocities $v_C(t,y)$ used in the 1DC model and to derive $\bar{v}_{\mathrm{C}}(t)$ were locally rotated using alongshore-varying shorenormal angles estimated from the land mask in the grid.

The wave energy term in (S3), E, was determined using

$$E(t) = \frac{1}{16}\rho g H_s(t)^2, \tag{S4}$$

where g is gravitational acceleration, ρ is the mean seawater density, and H_s is the significant wave height.

The standard deviation of the velocity vector $\sigma_{\vec{u}}$ in (3) can be written out as a function of H_s at the 5-m isobath. By definition, $H_s = 4\sigma_\eta$, where the σ_η is the standard deviation of the sea surface height. Orbital velocities and sea surface elevation of shallow water gravity waves have the same frequency, so $\sigma_{\vec{u}}$ is proportional to σ_η by a scale factor of $\sqrt{\frac{g}{h}}$ to change the dimension. The resulting expression for $\sigma_{\vec{u}}$ is,

$$\sigma_{\vec{u}}(t) = \sqrt{\frac{g}{h_{5\mathrm{m}}}}\frac{H_{s,5\mathrm{m}}(t)}{4}, \tag{S5}$$

where h is the constant depth of the water column. $H_{s,5\mathrm{m}}$ can be estimated from the significant wave height at the offshore location of the wave buoy, $H_{s,\mathrm{WB}}$ using Snell's Law and the conservation of wave energy flux given the difference in water depths. For this data set, $H_{s,5\mathrm{m}} = 0.88H_{s,\mathrm{WB}}$ on average. Combining (2), (3), (S2), and (S5) gives the following equation for $v_{1\mathrm{D}}$,

$$v_{1\mathrm{D}}(t) = -\frac{8}{3L\rho C_{\mathrm{D}}}\sqrt{\frac{2h_{5\mathrm{m}}}{\pi g}}\frac{S_{xy}(t)}{H_{s,5\mathrm{m}}(t)}, \tag{S6}$$

where C_{D} has flexibility as a fitting parameter, calculated using a simple linear regression (with intercept fixed to zero) between the wave-estimated velocity and $\bar{v}_{\mathrm{C}}$.

Calibrating C_d and velocity fit

Calibration of v_{1D} was done by fitting C_d using a linear regression with $\bar{v}_{\mathrm{C}}$ for the tuning period to a slope of 1 with no intercept (Fig. S1c). The resulting v_{1D} had strong agreement (R> 0.8) with $\bar{v}_{\mathrm{C}}$ (Fig. S1). The drag coefficient fit value was $C_{\mathrm{D}} = 0.004$, consistent with the value of 0.0033 found for the surf zone in Feddersen[3]. The resulting wave-driven v_{1D} captured the time variations in $\bar{v}_{\mathrm{C}}$ (Fig. S1b). During the biggest southerly waves in winter (spikes between Jan 1 and Mar 1 in Fig. S1b), v_{1D} overestimated $\bar{v}_{\mathrm{C}}$.

Historic wave forcing was used to compare the 1D model with water samples, which required recalibrating C_{d}. As before, C_{d} was used as a fitting parameter between alongshore velocity estimated from wave observations at the Imperial Beach Nearshore Buoy 155 managed by Coastal Data Information Program at Scripps Institution of Oceanography (SIO) and velocity measured by an acoustic Doppler current profiler (ADCP) deployed near Imperial Beach by the Coastal Processes Group at SIO. Velocities were measured from November 18, 2019 to December 4, 2019 at 16 Hz at 32.57291 N, -117.13597 E. To identify alongcoast currents, velocities were smoothed with an hour-long moving average, then tidally-filtered,[5] and finally rotated to the principal axis.[6] Waves were observed half-hourly at the 21 m isobath at 32.56968 N, -117.16895 E. Data from Buoy 155 is available during twelve deployments from November 2, 2007 to current day as of writing, with the sixth buoy deployment from August 24, 2018 to March 20, 2020 overlapping the full ADCP deployment. Correlation of measured and wave-estimated velocities was R = 0.34, consistent with previous wave model performance near Imperial Beach.[7] The tuned drag coefficient was $C_{\mathrm{d}} = 0.004$, consistent with the model-model fit in this study and previous literature.[3]

Willmott Skill Score

Willmott Skill Score (WSS) is a comprehensive model agreement metric that scales the mean square error by the potential error for a data set,[8]

$$\mathrm{WSS} = 1 - \frac{\Sigma_{i=1}^{i=N}(m_i - o_i)^2}{\Sigma_{i=1}^{i=N}(|m_i - \langle o \rangle| + |o_i - \langle o \rangle|)^2} \tag{S7}$$

where m is the 1D model value, o is the SD Bight model value, and N is the number of data points. WSS ranges from 0 to 1, with 1 being best.

Impact of Neglecting Alongshore Diffusivity

The 1D model equation used here (1) did not include alongshore diffusivity, unlike similar 1D models of nearshore alongshore advection.[1,9] This is because numerical alongshore diffusivity arising from the upwind advection scheme provided adequate alongshore diffusivity expected for this environment. The numerical alongshore diffusivity, K^*_{yy}, was estimated using a scale analysis,

$$K^*_{yy} \approx \frac{V\Delta y}{2} \tag{S8}$$

where $\Delta y = 30$ m was the grid cell length and $V = 0.1$ ms^{-1} was a typical velocity scale, chosen to be the RMS of $\bar{v}_{\mathrm{C}}$ as before. For this 1D model, the numerical $K^*_{yy} = 1.5$ m^{2}s^{-1}. Estimation of expected alongshore diffusivity follows Spydell et al.[10], who calculated nearshore alongshore diffusivity using drifters at Huntington Beach, CA and Torrey Pines, CA over a nearshore domain which extended beyond the surf zone to an offshore distance of 160 m. Spydell et al.[10] used two scaling estimates of K_{yy}. The first calculation used mixing length arguments,[11]

$$K_{yy} \approx \gamma V L, \tag{S9}$$

where γ is a fitting parameter, found in Spydell et al.[10] to be $\gamma = 0.52 \pm 0.08$. The second calculation used shear dispersion in a pipe,[12,13]

$$K_{yy} \approx V^2 T_0, \tag{S10}$$

where T_0 is the timescale of mixing, found in Spydell et al.[10] to be $T_0 = 154 \pm 13$ s. Using V and L in this study results in K_{yy} estimates of 10 and 1.5 $\mathrm{m^2 s^{-1}}$ for the mixing length and pipe shear dispersion arguments, respectively. This range is consistent with the range of $K_{yy} = 1 - 10$ $\mathrm{m^2 s^{-1}}$ estimated in Grimes et al.[1]. Grant et al.[9] found significantly higher estimates of $K_{yy} = 40 - 80$ $\mathrm{m^2 s^{-1}}$ in their field observations at Huntington Beach, CA, but Grant et al.[9] considered only the well-mixed region of the surf zone extending to 50 m offshore. The numerical diffusivity K^*_{yy} falls within the range of expected alongshore diffusivity found here, $K_{yy} = 1.5 - 10$ $\mathrm{m^2 s^{-1}}$. Inclusion of additional prescribed alongshore diffusivity was tested using K_{yy} ranging from 1 to 10 $\mathrm{m^2 s^{-1}}$, but model performance metrics varied by at most 3% of their original values. This justified neglecting additional alongshore diffusivity beyond numerical alongshore diffusivity.

Example data from binary analysis

The binary analysis converted tracer concentrations from the 1D and SD Bight models to Boolean using a tracer threshold. The two Boolean data series in time and y were then compared to generate a data series of True Positives, True Negatives, False Positives, and False Negatives. These four conditions were normalized as fractions of all time steps as a function of y. Example values from the alongshore location of four beaches, listed from most northern (HdC) to most southern (PTJ), are in Table S1. At all locations, True Negatives make up a majority of time steps and the fraction of True Negatives increases with y. The largest fraction of True Positives is found at Playas Tijuana (PTJ), comprising 15.42% of time steps.

Table S1: Example data comparing Boolean dye threshold exceedance between 1D model and SD Bight model at four alongshore locations corresponding to public beaches (locations illustrated in Fig. 1).

Location	% True Positive	% False Positive	% True Negative	% False Negative
HdC	0.72	2.56	95.84	0.89
SS	3.75	3.02	91.37	1.85
IB	6.79	6.14	82.54	4.53
PTJ	15.42	7.90	69.21	7.48

To further interpret these results, the Sensitivity and Specificity were calculated. Sensitivity > 0.6 for all beaches except HdC, the northernmost beach, and Specificity > 0.9 for all beaches (Table S1), improving slightly with y.

Table S2: Sensitivity and Specificity of 1D model predictions of Boolean threshold exceedence of SD Bight model dye at four alongshore locations corresponding to public beaches (locations illustrated in Fig. 1).

Location	Sensitivity	Specificity
HdC	0.45	0.97
SS	0.67	0.97
IB	0.60	0.93
PTJ	0.67	0.90

Comparison of 1D model to microbial source tracking

Nearshore 1D model prediction of two SADB WTP microbial source tracking sampling campaigns from Zimmer-Faust et al.[14] were evaluated with a best linear fit in log space, found by minimizing squared error between log model dye to log DNA copies. Slopes, intercepts, and correlation coefficients (R^2) are given below for three genetic markers, HF183, Lachno3, and *Enterococcus*, by sampling campaign. Sensitivity and Specificity were calculated using the BAC threshold for model dye and a 1 copy/mL threshold for genetic marker. Since nondetects were rare, sampling campaigns were consolidated in Sensitivity and Specificity

calculations. *Enterococcus* was detected in every sample, so no Specificity could be calculated. Consistent detection of *Enterococcus* may be because *Enterococcus* can also originate from animal and environmental sources, unlike HF183 and Lachno3 which are human-specific indicators.

Table S3: Data on the best linear fit between log of nearshore model prediction and log of Zimmer-Faust et al.[14] microbial sampling for three water quality indicators in two campaigns ("C1" = first sampling campaign on Oct 2–4, "C2" = second sampling campaign on Oct 27–29). Sensitivity and Specificity calculated using detects and nondetects per indicator for both C1 and C2.

Indicator	Slope (C1, C2)	Intercept (C1, C2)	R^2 (C1, C2)	Sensitivity	Specificity
HF183	8.3, 3.5	52, 29	0.42, 0.40	0.94	0.42
Lachno3	5.0, 3.6	33, 30	0.21, 0.39	0.82	0.22
Enterococcus	3.8, 2.6	29, 26	0.18, 0.54	0.82	N/A

Supporting Information Figures

Figure S1: a) SD Bight model alongshore-varying alongshore velocity, v_{C}, as a function of time during tuning period and y, with alongshore beach locations on right side (compare with Fig. 1). b) Time series of $\bar{v}_{\mathrm{C}}$ (black) with v_{1D} (blue), c) scatter plot of hourly $\bar{v}_{\mathrm{C}}$ vs v_{1D}, best fit line (black dashed line) has slope = 1.02, intercept = −0.0022, and R = 0.89. One-to-one line (magenta) for comparison with best fit in c). RMS of $\bar{v}_{\mathrm{C}}$ is 0.1 m s^{-1}.

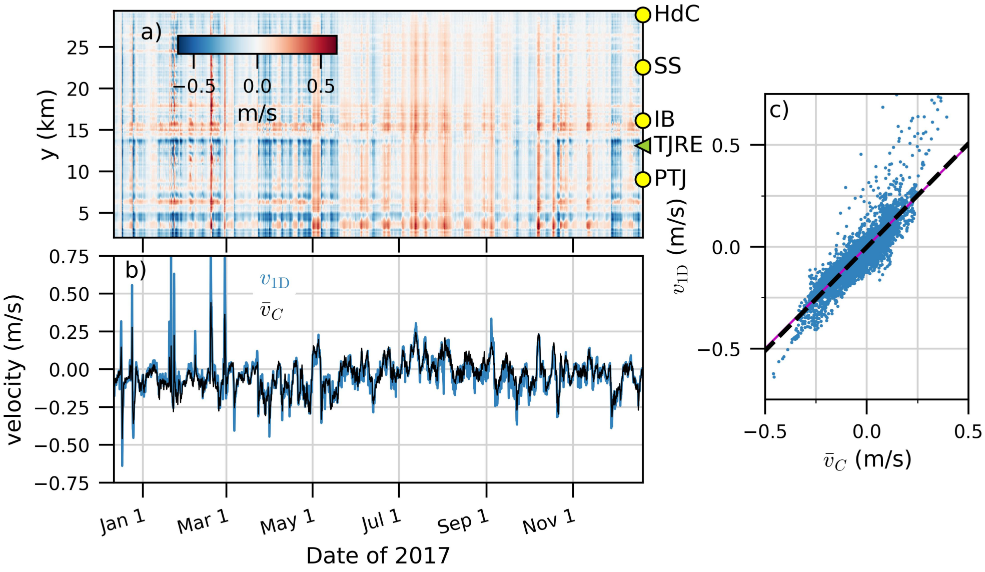

Figure S2: Dye concentrations at Imperial Beach (yellow circle labelled IB in Fig. 1) over three late summer months during model evaluation period from the SD Bight model (black solid line) and 1D model (blue solid line). The dashed red line indicates $C_{\text{BAC}} = 5 \times 10^{-4}$. Colored bars at top of figure depict True Positive, (purple), False Positive (orange), False Negative (blue), or True Negative (white). Four conditions defined in manuscript text.

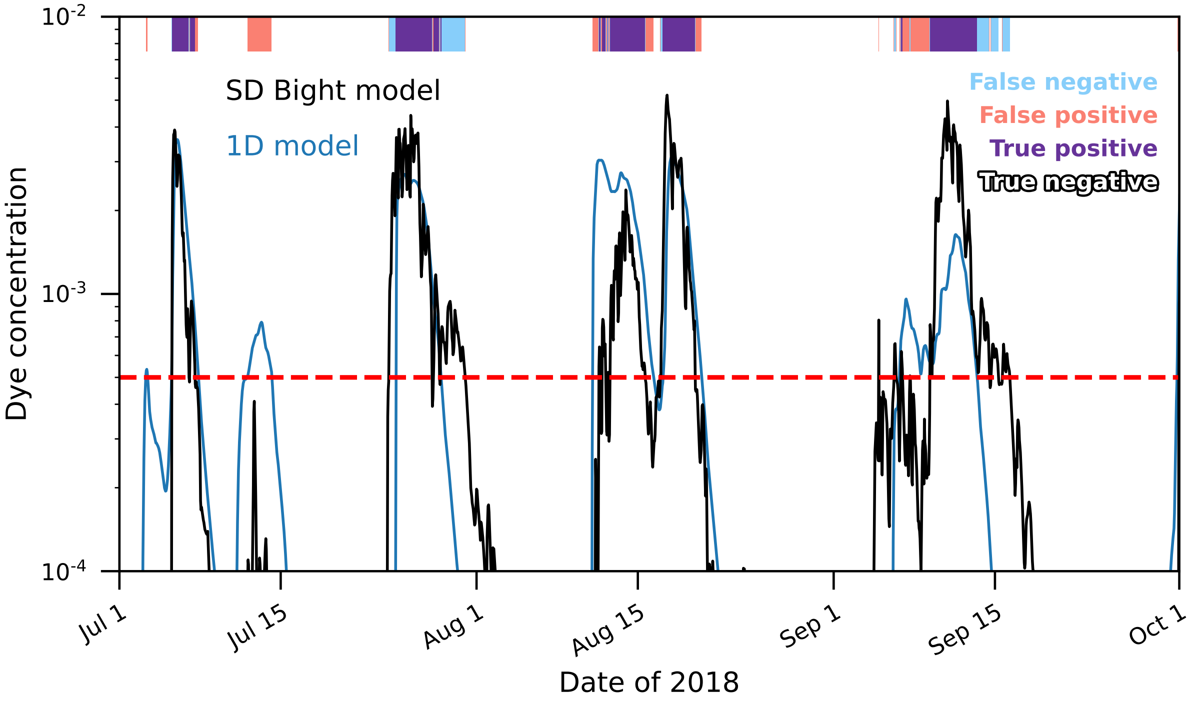

References

(1) Grimes, D. J.; Feddersen, F.; Giddings, S. N. Long-distance/time surf-zone tracer evolution affected by inner-shelf tracer retention and recirculation. *J. Geophysical Research Oceans* **2021**, *126*, e2021JC017661.

(2) Longuet-Higgins, M. S. Longshore currents generated by obliquely incident sea waves, parts 1 and 2. *J. Geophys. Res.* **1970**, *75*, 6778–6801.

(3) Feddersen, F. Weakly nonlinear shear waves. *J. Fluid Mech.* **1998**, *372*, 71–91.

(4) Ruessink, B. G.; Miles, J. R.; Feddersen, F.; Guza, R. T.; Elgar, S. Modeling the alongshore current on barred beaches. *J. Geophys. Res.* **2001**, *106*, 22,451–22,463.

(5) Godin, G. *The analysis of tides*; University of Toronto Press, 1972.

(6) Emery, W.; Thompson, R. *Data Analysis Methods in Physical Oceanography*; Pergamon, 2001.

(7) O'Reilly, W. C.; Olfe, C. B.; Thomas, J.; Seymour, R. J.; Guza, R. T. The California coastal wave monitoring and prediction system. *Coastal Engineering* **2016**, *116*, 118–132.

(8) Willmott, C. J. On the validation of models. *Physical Geography* **1981**, *2*, 184–194.

(9) Grant, S. B.; Kim, J. H.; Jones, B. H.; Jenkins, S. A.; Wasyl, J.; Cudaback, C. Surf zone entrainment, along-shore transport, and human health implications of pollution from tidal outlets. *J. Geophys. Res.* **2005**, *110*, C10025.

(10) Spydell, M. S.; Feddersen, F.; Guza, R. T. Observations of Drifter Dispersion in the Surfzone: The Effect of Sheared Alongshore Currents. *J. Geophys. Res.* **2009**, *114*, C07028.

(11) Tennekes, H.; Lumley, J. L. *A First Course in Turbulence*; MIT Press, 1972.

(12) Taylor, G. Dispersion of soluble matter in solvent flowing slowly through a tube. *Proc. Roy. Soc. A* **1953**, *219*, 186–203.

(13) Spydell, M. S.; Feddersen, F.; Guza, R. T.; Schmidt, W. E. Observing Surfzone Dispersion with Drifters. *J. Phys. Ocean.* **2007**, *27*, 2920–2939.

(14) Zimmer-Faust, A. G.; Steele, J. A.; Xiong, X.; Staley, C.; Griffith, M.; Sadowsky, M. J.; Diaz, M.; Griffith, J. F. A combined digital PCR and next generation DNA-sequencing based approach for tracking nearshore pollutant dynamics along the southwest U.S/Mexico border. *Front. Microbiol.* **2021**, *12*, 674214.
